# Conditioned pain modulation and nociception measured by pain-related evoked potentials in patients with polyneuropathy

**DOI:** 10.3389/fneur.2026.1776830

**Published:** 2026-04-02

**Authors:** Laura Josephine Bubenzer, Julia Jessen, Martin Tegenthoff, Oliver Höffken, Johannes Forsting, Andrea Westermann, Andreas Schwarzer, Elena K. Enax-Krumova, Özüm Simal Özgül

**Affiliations:** 1 Department of Neurology, BG University Hospital Bergmannsheil Bochum gGmbH, Ruhr University Bochum, Bochum, Germany; 2 Department of Pain Medicine, BG University Hospital Bergmannsheil Bochum gGmbH, Ruhr University Bochum, Bochum, Germany

**Keywords:** conditioned pain modulation, endogenous pain inhibition, mixed fiber neuropathy, pain-related evoked potentials, polyneuropathy, small fiber neuropathy

## Abstract

**Objective:**

Aim of the study was to assess alterations in nociception and conditioned pain modulation (CPM) in polyneuropathy (PNP) patients depending on the presence of pain and the pattern of affected nerve fibers.

**Methods:**

Pain-related evoked potentials (PREP) after painful cutaneous electrical stimulation (PCES) were recorded in 44 PNP patients and 19 healthy controls (HC) with stimulation via concentric surface electrodes. Using the same electrodes for electrical stimulation as test stimulus (TS) and cold water as the conditioned stimulus (CS), we additionally assessed the CPM effects based on changes in pain intensities and amplitudes of evoked potentials elicited by PCES. Subgroup analysis included painless, painful, small (SFN), mixed (MFN), and large (LFN) fiber neuropathies.

**Results:**

During the PREP procedure, patients required higher stimulus intensities compared to HC and reported stronger pain. SFN patients had the highest pain scores despite similar stimulus intensities as in HC. N1-latencies of PREP were longer in LFN, N1P1-amplitudes of PREP did not differ. During the CPM testing, the CS induced a significant decrease in pain ratings in HC and painful PNP. The CPM-effects were similar between groups. N1P1-amplitudes for PCES were lower in patients throughout the CPM assessment. Patients with painful MFN and SFN required lower TS-intensities.

**Discussion:**

The CPM effects in painful PNP and HC were similar, despite hints for sensitization in CPM and PREP-parameters, especially in small fiber involvement. Amplitude changes during CPM-procedure were independent of pain. Deafferentation influences both PREP and CPM parameters, such as N1-latencies or N1P1-amplitudes, but not the CPM-effect itself.

## 1 Introduction

Polyneuropathy (PNP) is characterized by sensory symptoms such as numbness, pain or paresthesia, usually located in the distal limbs (1). Large, mixed, and small fiber neuropathy (LFN, MFN, SFN) can be differentiated based on clinical symptoms, electrophysiology, and other diagnostic measures (2). Symptoms may vary depending on the nerve fibers predominantly affected (3). Some patients also report pain. To this date, the mechanisms that lead to neuropathic pain in PNP are still not fully understood. However, patients with painful SFN seem to suffer from more severe pain than patients with painful MFN (4).

Diagnosing PNP relies on anamnesis, clinical neurological examination, nerve conduction studies (NCS), and electromyography (EMG) (5). Furthermore, quantitative sensory testing (QST) and intra-epidermal nerve fiber density (IENFD) examination are recommended for detecting small fiber impairments (4, 6). QST profiles can differ between painful and painless PNP, although sensory loss is equally prevalent in patients with and without spontaneous pain (7). Laser- (LEP) or contact-heat-evoked potentials (CHEP) represent alternatives for assessing the nociceptive Aδ- and C-fiber transmission (8, 9). The generation of Pain-related evoked potentials (PREP) using superficial planar concentric electrodes for electrically stimulating nociceptive fibers is a further option for investigating small fiber transmission (10) and appears to be additionally useful for detecting SFN (5). The small anode–cathode distance of the concentric electrode allows a selective stimulation of free nerve endings, especially Aδ-fibers, in the superficial layer of the dermis (11). During stimulation at the two-fold of the individual pain threshold (PT), an electroencephalogram (EEG) records the elicited potentials over Cz, allowing the assessment of N1-latencies and peak-to-peak amplitudes (N1P1-amplitudes). Previous studies on PREP have already shown that PNP patients show prolonged latencies and reduced amplitudes (12–15), for review see (16).

Conditioned pain modulation (CPM) assesses the endogenous pain modulation in humans, examining the analgesic effect of a noxious conditioned stimulus (CS) on a noxious test stimulus (TS) (17). CPM can be performed using various noxious stimuli, like cold, heat, laser or electrical stimulation (18). Recently, we have introduced a novel CPM model that utilizes nociceptor stimulation and the generation of corresponding cortical potentials following electrical stimulation as TS using a concentric electrode and a cold-water bath as CS, enabling the evaluation of the CPM-effect based on objective electrophysiological measurements (19, 20). Impaired endogenous pain modulation appears to be associated with chronic pain development (21–23). However, only a few studies investigated the CPM-effect in PNP patients and especially in patients with painful PNP (24–26).

Our study aimed to investigate whether pain modulation and nociception differ between patients with PNP, especially in painful PNP, compared to healthy controls, and whether these differences depend on the type of affected nerve fibers. Furthermore, we expected patients with MFN and SFN to show prolonged latencies and reduced amplitudes compared to patients with LFN due to the selective activation of the small fibers in PREP. We hypothesized that patients with affected nociceptive fibers (Aδ, C) show a reduced CPM-effect compared to patients without nociceptive fiber affection due to reduced peripheral input to induce the CPM-effect. In addition, we examined associations between PREP and clinical variables such as pain intensity and disease duration. Assuming that PREP would reflect the extent of small-fiber damage, as previously suggested (27, 28), we specifically analyzed the relationship between PREP and CPM to determine whether reduced peripheral input due to PNP is linked to a diminished CPM-effect. Since previous studies showed that the CPM-effect is less efficient in patients with pain, we also hypothesized that the CPM-effect in patients with painful PNP is less efficient than in painless PNP patients and healthy controls.

## 2 Materials and methods

### 2.1 Study subjects

Patients with confirmed PNP meeting the inclusion criteria and consenting to participate were recruited between June 2020 and March 2024 from patients of the University Hospital Bergmannsheil Bochum, Germany. Inclusion criteria were as follows: (1) paresthesia or dysesthesia or pain in arms or legs and/or (2) abnormal NCS and/or (3) abnormal QST indicating small and/or large fiber sensory loss and/or (4) reduced IENFD in skin punch biopsy (if performed). Healthy controls older than 18 years were recruited among relatives, friends, and hospital visitors. Based on Giertmühlen et al. (2015), healthy subjects had to be pain-free, show normal results in NCS and QST, have no medical conditions, and have no medication intake (29). Exclusion criteria for both groups included age <18 years, communication issues, topical lidocaine or capsaicin use, epilepsy, psychiatric disorders, severe affection of the central nervous system, other neurological or painful diseases, peripheral artery disease, severe internal disease, substance abuse and pregnancy. In healthy controls, a PNP diagnosis or any form of pain were additional exclusion criteria. Parameters such as age, sex, height, duration of illness, comorbidities, presence of pain, medication intake and affected areas, were recorded to be able to evaluate their potential correlations with the primary outcome measures.

### 2.2 Study design

The study was approved by the local ethics committee of the Faculty of Medicine, Ruhr-University Bochum, Germany (Reg. No. 18-6629-BR). All participants gave written informed consent. The study was conducted at the Department of Neurology, University Hospital Bergmannsheil Bochum, Germany. Each participant attended one session, which lasted 3 h, including informed consent, neurological examination, NCS, QST, PREP, and CPM. The order was not randomized; CPM always followed PREP. If both NCS and QST results were normal and patients did not have any relevant symptoms, they were excluded from the study.

### 2.3 NCS

NCS was performed using the in-house protocol for PNP screening. Motor studies included the right peroneal and tibial nerves. Sensory studies included the right radial and left sural nerves. Distal motor latencies, amplitudes, and sensory/motor conduction velocities were assessed using a Neurowerk four-channel EMG device (esumedics GmbH). The reference values used were those by Stöhr et al. (30). NCS was rated abnormal if ≥2 of the four nerves were affected.

### 2.4 QST

QST was conducted at the Department of Pain Medicine by a trained investigator following a standardized protocol of the German Research Network on Neuropathic Pain (31, 32). Testing was performed on the dorsum of both feet after familiarization with the procedure on the dorsum of the hand. Following parameters were assessed: cold and warm detection thresholds (CDT/WDT), detection of temperature changes (TSL), cold and heat pain thresholds (CPT/HPT), paradoxical heat sensation (PHS), pressure pain threshold (PPT), mechanical pain sensitivity (MPS), mechanical detection threshold (MDT), mechanical pain threshold (MPT), vibration detection threshold (VDT), wind-up-ratio for muscular pressure pain (WUR) and dynamical mechanic allodynia (DMA). CDT, WDT, TSL, CPT, HPT, and PHS were measured using a Medoc® thermotester, which administers computer-controlled temperature stimuli. MDT was determined using a set of standardized von Frey filaments “OptiHair2”®. MPT was measured using standardized needle stimulus simulators. Needle stimulus simulators were also used to assess MPS and WUR. DMA was determined by alternating stimulation with a cotton swab, brush, or cotton ball. First, the test subjects assessed the pain intensity of a single stimulus and then compared it with the intensity of a series of 10 stimuli. VDT was determined using a standardized tuning fork C 128 Hz/C 64 Hz according to Ryder-Seiffer. The PPT was assessed using a pressure algometer from Wagner®. Z-scores for each QST item (except for PHS and DMA) were calculated based on the published reference values (32). A negative z-score indicated loss of sensation <−1,96, and gain of sensation was indicated by a positive z-score>1,96.

### 2.5 PREP

**Figure 1:**
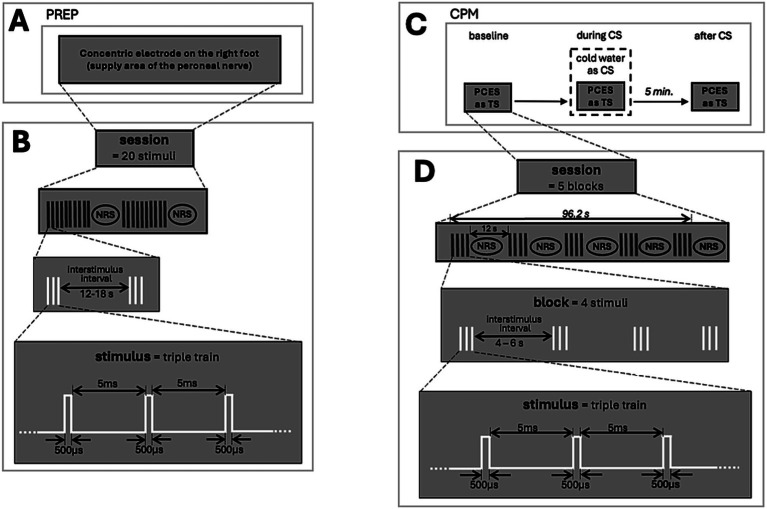
Experimental procedure and stimulation paradigm. **(A)** Timeline of PREP recordings, **(B)** stimulation paradigm of PREP, **(C)** timeline of CPM protocol, and **(D)** stimulation paradigm of CPM. PREP, pain-related evoked potentials; NRS, Numeric Rating Scale; CPM, conditioned pain modulation; CS, conditioned stimulus; TS, test stimulus; PCES, painful cutaneous electrical stimulation.

Figures 1A,B shows the experimental setup and stimulation paradigm. Testing was conducted in a quiet, temperature-controlled room. Participants sat semi-reclined, instructed to relax and avoid movement. Electrical stimulation was applied using a superficial planar concentric electrode as previously described (10), placed on the dorsum of the right foot (supply area of the peroneal nerve). Due to its concentric structure and the small distance between the anode and cathode, the electrode can generate a high current density even at low current intensities. This leads to selective depolarization of superficial dermal layers innervated by nociceptive A-delta fibers, while deeper layers containing A-beta fibers remain unaffected. Detection threshold (DT) and PT were determined by gradually increasing/decreasing current intensities starting with 0.2 mA steps until a sensation (for DT) or a pinprick-like pain (for PT) was reported. Afterwards, 20 triple pulses (3 successive monopolar square waves; duration 500 μs; interwave interval within the triple-train 5 ms; interstimulus interval: 12–18 s) were applied at the two-fold intensity of the individual PT. For pain assessment, the participants were asked to rate the pain intensity using a numerical rating scale (NRS; 0 = no pain, 100 = strongest pain imaginable) after every 10 stimuli. PREPs were recorded using subcutaneous needle electrodes above Cz referred to linked earlobes (A1-A2) of the international 10–20 systems and stored for offline analysis with a 32-channel amplifier (Brain Amp, Brain Products, Germany; Bandwidth: 1 Hz–1 kHz; digitization sampling rate: 2.5 kHz). An examiner visually analyzed the evoked potentials in sweeps ranging from 200 ms before to 800 ms after PREP stimulus onset using Vision Recorder Version 1.03. Following previous studies (10–14, 33), the first sweep was rejected to avoid bias by the initial starting response. The evoked potential is the sum potential of the 19 applied stimuli. For further analysis, the mean value of the two pain ratings after every 10 stimuli, as well as the DT, PT, and stimulation intensity, were assessed. Furthermore, we also evaluated N1-latencies and N1P1-amplitudes.

### 2.6 CPM

Figures 1C,D shows the experimental setup and stimulation paradigm. The CPM procedure followed previous protocols (19, 20, 34), using painful cutaneous electrical stimulation (PCES) on the right hand (supply area of the radial nerve) as TS and the immersion of the left hand in a 10 °C cold-water bath as CS. PCES was applied using the same concentric electrode that was used for PREP. The hand was chosen as the test area as it is less frequently affected by PNP. To obtain a sufficiently painful stimulus as TS, we selected a stimulation intensity of 60 on the NRS for PCES-EP, as previously recommended (35). The intensity of the TS was determined by increasing the electric current in steps of 0.2 mA until the participants reported pain intensity of 60 on the NRS from 0–100. The determined TS was applied during the CPM procedure as TS_baseline_ (before CS), TS_during_ (during CS), and TS_after_ (after CS). The cold-water bath was applied for 96 s. At baseline, 20 triple pulses (interstimulus interval: 4–6 s) were applied as TS, and participants were asked to rate the induced pain after every block consisting of four stimuli. For CS, the participants placed their left hand in the cold-water bath, and after 20 s, the TS was applied again. Participants were asked to rate the electrically induced pain after every block and the cold-water pain separately. Five minutes after removing the hand from the cold-water bath, TS was applied again, and the pain intensity was determined equally to TS_baseline_.

For the calculation of the CPM-effect_PAIN_ and the CPM-effect_AMPLITUDE_ we followed recommendations of a previous protocol (19). The parallel CPM-effect_PAIN_ was calculated as the difference between the mean of pain ratings during CS and the mean of pain ratings at baseline, and the sequential CPM-effect_PAIN_ as the difference between the mean of pain ratings after CS and the mean of pain ratings at baseline. We regarded a CPM-effect_PAIN_ < 0 as a hint for efficient pain modulation.

The parallel CPM-effect_AMPLITUDE_ was calculated as the quotient between the mean of the N1P1-amplitudes during CS and the mean of the N1P1-amplitudes at baseline, and the sequential CPM-effect_AMPLITUDE_ as the quotient between the mean of N1P1-amplitudes after CS and the mean of N1P1-amplitudes at baseline. The reason for a different calculation of the CPM-effect_AMPLITUDE_ compared to the CPM-effect_PAIN_ is that the magnitude of the amplitudes can vary greatly between individuals. To capture a relative change in amplitude size, the calculation was performed as the quotient of the two amplitudes. We regarded a CPM-effect_AMPLITUDE_ < 1 as a hint for efficient pain modulation.

### 2.7 Questionnaires

All patients were asked to fill in the following questionnaires to better characterize the clinical features of the study groups. They were screened for symptoms of SFN using the Small Fiber Neuropathy Screening List (SFNSL). Pain intensity was assessed using the Brief Pain Inventory (BPI) (36) and the Pain Detect Questionnaire (PD-Q), which additionally allows the discrimination between neuropathic and nociceptive pain components (37). Depression and anxiety symptoms were evaluated using the Patient Health Questionnaire-4 (PHQ-4), which is a self-report questionnaire that includes a 2-item depression scale and a 2-item anxiety scale (38).

### 2.8 Definition of subgroups

Based on QST, NCS, and skin punch biopsy, patients were classified into an LFN, MFN, and SFN group. SFN was defined by (1) a z-score <−1.96 for CDT and WDT or an abnormal PHS, but normal MDT and VDT, or (2) reduced IENFD in skin punch biopsy (if performed), and (3) no abnormal NCS. Patients were assigned to the LFN group only if the z-score for MDT or VDT was <−1.96, QST showed no other abnormalities, and NCS was abnormal. MFN was defined by combined small and large fiber involvement.

Further, we divided the patients into a painful and painless PNP group. The painless group included those without PNP-related pain since being diagnosed and did not report any other pain during the last 4 weeks.

For a detailed analysis of the CPM, only patients with pain were divided into subgroups (painful LFN, painful MFN, and painful SFN).

### 2.9 Statistical analysis

Statistical analysis was performed using IBM SPSS, version 29.0.2.0, with *p* < 0.05 considered significant. Descriptive statistics are given as mean and standard deviation or median and range. Normal distribution was assessed using the Kolmogorov–Smirnov-test. In the absence of normal distribution, we used non-parametric tests. The following PREP parameters were included in the analysis: DT, PT, stimulation intensity, pain rating, N1-latency, and N1P1-amplitude. The CPM parameters that were tested included NRS 60 rated current intensity, pain ratings of TS and CS, N1P1-amplitudes, parallel CPM-effect_AMPLITUDE_ and CPM-effect_PAIN_, and sequential CPM-effect_AMPLITUDE_ and CPM-effect_PAIN_. Mann–Whitney-U-tests compared PREP and CPM parameters between patients and healthy controls. Kruskal-Wallis-tests compared patient subgroups (LFN, MFN, SFN) and healthy controls, as well as painful PNP, painless PNP, and healthy controls. Spearman correlations examined associations between body height and N1-latency, duration of disease and N1-latency/N1P1-amplitude, *z*-values of CDT/WDT (both values display an Aδ- and C-fiber function), and N1-latency/N1P1-amplitude, average pain intensity in the last 4 weeks and N1-latency/N1P1-amplitude; and CPM-effects with disease duration, pain intensity, PREP N1-latency/N1P1-amplitude and painfulness of PREP. Friedman-test assessed CPM changes across TS_baseline_, TS_during,_ and TS_after_. Since we only conducted an exploratory analysis of the data, no multiple comparison correction was applied.

## 3 Results

### 3.1 Clinical data

**Figure 2:**
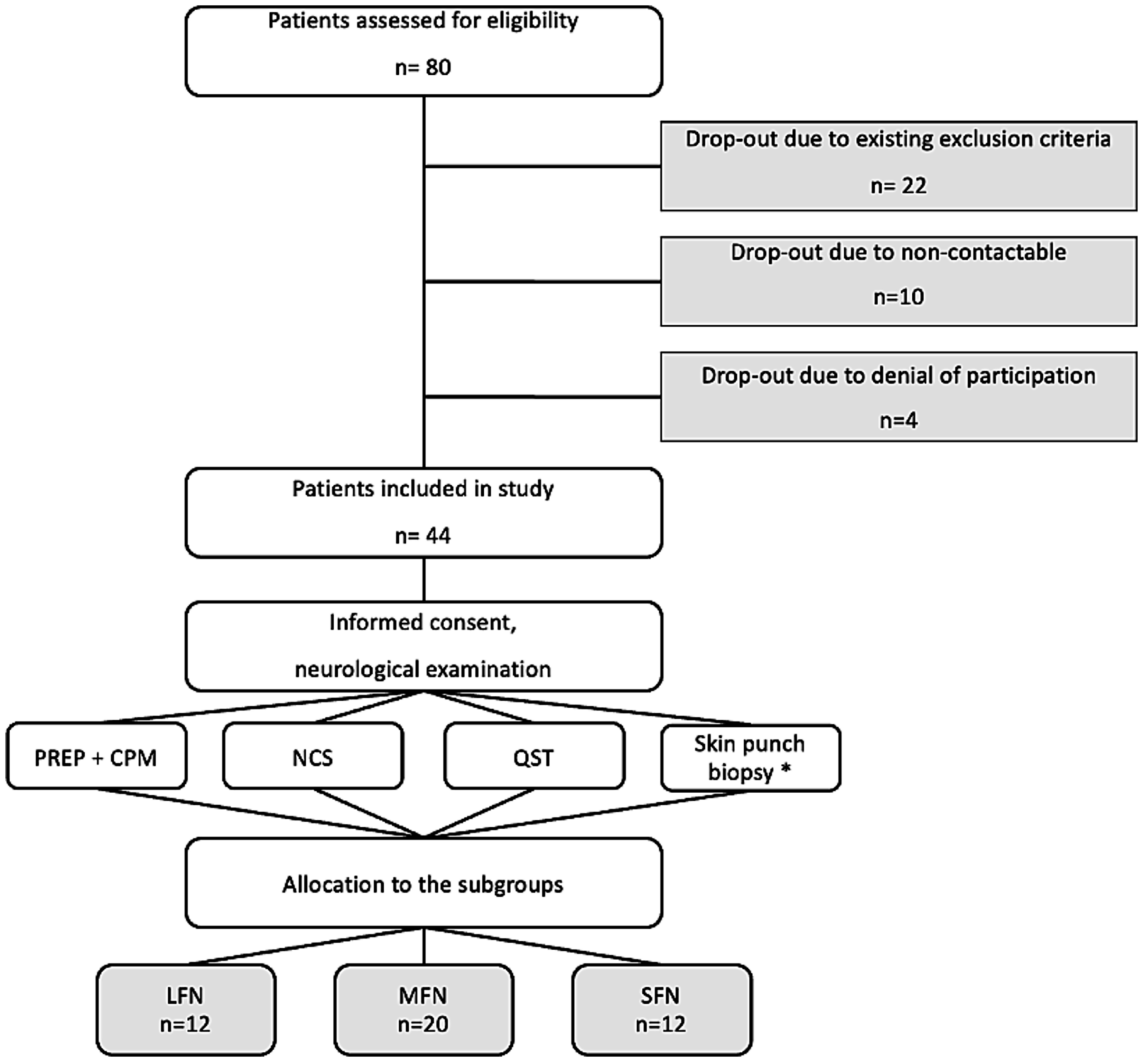
Flowchart of the patients’ recruitment and drop-outs. PREP, Pain-related evoked potentials; CPM, conditioned pain modulation; NCS, nerve conduction studies; QST, quantitative sensory testing; LFN, large fiber neuropathy; MFN, mixed fiber neuropathy; SFN, small fiber neuropathy. * A standard skin punch biopsy was not performed in all patients; the results of skin punch biopsy were only considered if they were performed as part of the clinical diagnostic workup.

The study included 44 PNP patients and 19 healthy controls. For analysis, patients were grouped into LFN (*n* = 12), MFN (*n* = 20), and SFN (*n* = 12), and later into painful (*n* = 34) and painless PNP (*n* = 10), as described in the Methods section (Figure 2). Gender distribution did not differ significantly, but patients were significantly older than healthy controls. The Kolmogorov–Smirnov-test showed that not all variables were normally distributed. Clinical and demographic data are presented in Table 1.

**Table 1:** Clinical and demographic data of patients with PNP and healthy controls.

Clinical and demographic data	Healthy controls	All patients	LFN	MFN	SFN
*n* (%)	19 (100%)	44 (100%)	12 (27%)	20 (45%)	12 (27%)
Sex [female, *n* (%)]	6 (32%)	18 (41%)	2 (17%)	8 (40%)	8 (67%)
Age (years), mean ± SD	45 ± 15	59.5 ± 11	58.3 ± 11	62.5 ± 10	55.7 ± 11
Height (cm), mean ± SD	175.74 ± 10.9	176.36 ± 10	180.83 ± 9.9	175.05 ± 10.5	174.08 **±** 8.4
Painful PNP, *n* (%)	-	34 (77%)	8 (67%)	17 (85%)	9 (75%)
Average pain intensity in the last 4 weeks, NRS (0–10), mean ± SD	-	4.2 ± 3	4 ± 3.5	4.5 ± 2.8	3.8 ± 2.9
Duration of disease, *n* (%)					
Up to 1 year	-	9 (20%)	2 (17%)	4 (20%)	3 (25%)
1 to 5 years	-	19 (43%)	4 (33%)	10 (50%)	5 (42%)
Over 5 years	-	16 (36%)	6 (50%)	6 (30%)	4 (33%)
Area affected, *n* (%)					
Feet	-	25 (57%)	7 (58%)	12 (60%)	6 (50%)
Hands	-	1 (2%)	-	-	1 (8%)
Hands and feet	-	18 (41%)	5 (42%)	8 (40%)	5 (42%)
Abnormal NCS, *n* (%)	-	28 (64%)	10 (83%)	18 (90%)	-
No sural nerve potential present, *n* (%)	-	12 (27%)	7 (58%)	5 (25%)	-
Sural nerve SNAP (mV)	13.65 ± 6.3	10.88 ± 7.7	6.06 ± 4.6	10.67 ± 9.9	13.13 ± 4.6
Sural nerve NCV (m/s)	47.67 ± 5.4	48.04 ± 9.2	52.78 ± 7.8	47.54 ± 11.8	46.7 ± 4.9
No peroneal nerve potential present, *n* (%)	-	14 (32%)	6 (50%)	7 (35%)	-
Peroneal nerve DML (ms)	3.62 ± 0.4	4.04 ± 0.9	4.06 ± 1	4.4 ± 1.2	3.68 ± 0.5
Peroneal nerve SNAP (mV)	6.98 ± 3.6	5.01 ± 3	5.17 ± 4.7	2.85 ± 1.7	7.08 ± 1.2
Peroneal nerve NCV (m/s)	46.93 ± 3.4	44.1 ± 4.9	41.98 ± 4.7	43.53 ± 5.4	45.73 ± 4.4
No tibial nerve potential present, *n* (%)	-	7 (16%)	3 (25%)	4 (20%)	-
Tibial nerve DML (ms)	4.81 ± 0.7	5.63 ± 1.6	5.83 ± 1.1	6.04 ± 2.1	4.93 ± 1
Tibial nerve SNAP (mV)	14.67 ± 4	9.09 ± 7.4	6.42 ± 7.1	7.24 ± 8.8	13.55 ± 2.4
Tibial nerve NCV (m/s)	44.68 ± 4	38.84 ± 7.3	35.23 ± 8.3	37.39 ± 7.6	43.47 ± 2.9
No radial nerve potential present, *n* (%)	-	-	-	-	-
Radial nerve SNAP (mV)	24.66 ± 7.9	16.46 ± 10.8	10.31 ± 7.8	14.82 ± 9.2	25.21 ± 10.9
Radial nerve NCV (m/s)	62.74 ± 4.5	57.74 ± 11	52.58 ± 11.4	58.79 ± 12.8	61.33 ± 4.5
QST – sensory gain, *n* (%)					
No gain	-	26 (59%)	7 (58%)	12 (60%)	7 (70%)
Thermal gain	-	-	-	-	-
Mechanical gain	-	16 (36%)	5 (42%)	6 (30%)	5 (42%)
Thermal and mechanical gain	-	1 (2%)	-	1 (5%)	-
Reduced intraepidermal nerve fiber density in the skin punch biopsy, *n* (%)					
Yes	-	13 (30%)	-	4 (20%)	9 (75%)
No	-	2 (5%)	-	1 (5%)	1 (8%)
Not performed	19 (100%)	29 (66%)	12 (100%)	15 (75%)	2 (17%)
Pain medication intake, *n* (%)					
No medication intake	19 (100%)	27 (61%)	6 (50%)	12 (60%)	9 (75%)
Classic analgesics (e.g., NSAID, metamizol)	-	8 (18%)	2 (17%)	4 (20%)	2 (17%)
Antidepressants	-	3 (7%)	-	2 (10%)	1 (8%)
Anticonvulsant	-	9 (20%)	3 (25%)	4 (20%)	2 (17%)
Opioids	-	6 (14%)	1 (8%)	4 (20%)	1 (8%)
Etiology, *n* (%)					
Idiopathic/unknown	-	29 (66%)	4 (33%)	13 (65%)	12 (100%)
Immune-modulated (e.g., CIDP)	-	9 (20%)	5 (42%)	4 (20%)	-
Toxic	-	1 (2%)	1 (8%)	-	-
Metabolic	-	1 (2%)	1 (8%)	-	-
Hereditary	-	4 (9%)	1 (8%)	3 (15%)	-
Comorbidities, *n* (%)					
Arterial hypertension	-	20 (45%)	6 (50%)	10 (50%)	4 (33%)
Cancer	-	5 (11%)	-	4 (20%)	1 (8%)
Respiratory disease	-	5 (11%)	1 (8%)	4 (20%)	-
Diabetes mellitus type II	-	4 (9%)	2 (17%)	1 (5%)	1 (8%)
Thyroid disease	-	4 (9%)	-	3 (15%)	1 (8%)
Coronary heart disease	-	3 (7%)	-	3 (15%)	-
Rheumatic disease	-	3 (7%)	-	3 (15%)	-
Cardiac arrhythmias	-	3 (7%)	-	2 (10%)	1 (8%)
History of alcohol abuse	-	2 (5%)	1 (8%)	1 (5%)	-
Hypovitaminosis, mineral deficiency	-	2 (5%)	1 (8%)	1 (5%)	-
Gout	-	2 (5%)	-	2 (10%)	-
Other	-	6 (14%)	2 (17%)	4 (20%)	-
None	-	12 (27%)	4 (33%)	2 (10%)	6 (50%)
Questionnaires					
Patient Health Questionnaire-4 (PHQ-4), mean ± SD	-	3.91 ± 3.23	4.17 ± 3.61	4.2 ± 3.41	3.17 ± 2.59
Small Fiber Neuropathy Screening List (SFNSL), *n* (%)					
Few or no SFN-related symptoms (score 0–11)	-	7 (16%)	3 (25%)	3 (15%)	1 (8%)
Possible SFN (score 12–47)	-	33 (75%)	8 (67%)	15 (75%)	10 (83%)
Probable SFN (score 48–84)	-	4 (9%)	1 (8%)	2 (10%)	1 (8%)
PainDetect (PD-Q), *n* (%)					
No pain	-	10 (23%)	4 (33%)	3 (15%)	3 (25%)
Nociceptive Pain (score < 12)	-	9 (20%)	3 (25%)	5 (25%)	1 (8%)
Possible Neuropathic Pain (score 13–18)	-	15 (34%)	3 (25%)	6 (30%)	6 (50%)
Probable Neuropathic Pain (score 19–38)	-	10 (23%)	2 (17%)	6 (30%)	2 (17%)
Brief Pain Inventory (BPI), mean ± SD	-	26.25 ± 14.71	25.42 ± 13.26	26 ± 15.16	27.5 ± 16.46

LFN, large fiber neuropathy; MFN, mixed fiber neuropathy; SFN, small fiber neuropathy; SD, standard deviation; PNP, polyneuropathy; NRS, Numeric Rating Scale; NCS, nerve conduction studies; SNAP, sensible nerve action potential; NCV, nerve conduction velocity; DML, distal motor latency; QST, quantitative sensory testing; NSAID, nonsteroidal anti-inflammatory drugs; CIDP, chronic inflammatory demyelinating polyneuropathy.

### 3.2 Nociception measured by PREP

#### 3.2.1 Patients vs. healthy controls

Table 2 presents PREP parameters and significant *p*-values. To account for multiple testing, a Bonferroni correction was applied blockwise, cut-off *p*-values are reported in Table 2. Results that remained statistically significant after blockwise correction are highlighted in bold in Table 2. Patients showed significantly higher DT, PT, stimulus intensities, and pain ratings, as well as prolonged N1-latencies compared to healthy controls. N1P1-amplitudes did not differ. Disease duration did not correlate with N1-latencies or N1P1-amplitudes, but N1-latencies correlated moderately with body height (*r* = 0.416, *p* = 0.008). No height difference was found between groups (*p* = 0.827).

**Table 2:** PREP parameters.

			Subgroups depending on the presence of pain		Subgroups depending on the affected nerves			
PREP parameters	Healthy controls (*n* = 19)	All patients (*n* = 44)	Painful (*n* = 34)	Painless (*n* = 10)	LFN (*n* = 12)	MFN (*n* = 20)	SFN (*n* = 12)	*p*-values
DT right (mA)	0.92 ± 0.45 (0.8; 0.4-2)	1.46 ± 1.07 (1.1; 0.2-4.5)	1.45 ± 0.99 (1.1; 0.2-4.5)	1.52 ± 1.37 (1.05; 0.3-4.5)	1.45 ± 1.04 (1.13; 0.3-3.5)	1.79 ± 1.27 (1.13; 0.5-4.5)	0.93 ± 0.37 (0.9; 0.2-1.7)	AP vs. HC: 0.042, **MFN vs. HC: 0.008**, SFN vs. MFN: 0.036, PF vs. HC: 0.036
PT right (mA)	1.39 ± 0.72 (1.2; 0.4-3)	2.95 ± 3.59 (1.9; 0.5-22)	2.98 ± 3.84 (1.8; 0.9-22)	2.85 ± 2.74 (2; 0.5-10)	2.97 ± 3.33 (1.75; 0.5-10)	3.66 ± 4.59 (2.45; 1-22)	1.75 ± 0.62 (1.6; 0.9-3)	**AP vs. HC: 0.003,** **MFN vs. HC: <0.001,** **PF vs. HC: 0.005,** PL vs. HC: 0.025
Stimulation intensity right at 2-fold PT (mA)	2.79 ± 1.44 (2.4; 0.8-6)	5.9 ± 7.18 (3.8; 1-44)	5.96 ± 7.68 (3.6; 1.8-44)	5.7 ± 5.48 (4; 1-20)	5.94 ± 6.66 (3.5; 1.0-20)	7.32 ± 9.18 (4.9; 2-44)	3.5 ± 1.25 (3.2; 1.8-6)	**AP vs. HC: 0.003,** **MFN vs. HC: <0.001,** **PF vs. HC: 0.005,** **PL vs. HC: 0.025**
Pain rating right (NRS)	24.5 ± 25.17 (13; 1-90)	38.7 ± 20.52 (42.5; 5.0-82.5)	41.04 ± 19.47 (42.5; 10-82.5)	30.75 ± 23.04 (27.5; 5-72.5)	33.21 ± 25.34 (22.5; 5-65)	37.1 ± 19.67 (41.25; 10-82.5)	46.88 ± 15 (47.5; 22.5-72.5)	**AP vs. HC: 0.005,** MFN vs. HC: 0.029, **SFN vs. HC: 0.002,** **PF vs. HC: 0.002**
N1 latency right (ms)	174.4 ± 54.5 (171; 94-262)	211.2 ± 54.1 (189; 102-334)	211.6 ± 54.8 (190; 102-334)	210.2 ± 54.6 (186; 126-291)	233.5 ± 40.8 (230; 182-296)	226.3 ± 56.5 (190; 160-334)	169.5 ± 38.3 (173; 102-260)	AP vs. HC: 0.026, **MFN vs. SFN: 0.004,** **MFN vs. HC: 0.007,** **SFN vs. LFN: 0.002,** **LFN vs. HC: 0.004,** PF vs. HC: 0.038
N1P1 amplitude right (μV)	24.67 ± 9.53 (22.69; 10.99-40.83)	24.02 ± 9.8 (21.84; 10.79-56.94)	25.17 ± 10.53 (23.95; 10.79-56.94)	20.57 ± 6.41 (20.62; 11.78-35.34)	22.32 ± 6.83 (23.42; 10.79-33.14)	21.68 ± 7.01 (20.68; 11.75-36.41)	28.89 ± 13.77 (25.4; 11.33-56.94)	

Given are the mean value and the standard deviation as well as the median and the minimum to maximum. To control for multiple comparisons, a Bonferroni correction was applied. The significance level was adjusted to *p* < 0.0167. Results that remained statistically significant after correction are highlighted in bold. LFN, large fiber neuropathy; MFN, mixed fiber neuropathy; SFN, small fiber neuropathy; AP, all patients; HC, healthy controls; PF, painful; PL, painless; DT, detection threshold; PT, pain threshold; NRS, Numeric Rating Scale.

#### 3.2.2 Comparison between painful, painless subgroups and healthy controls

In patients with painful PNP, DT was significantly higher than in healthy controls. PT and stimulation intensities were higher in painful and painless PNP compared to healthy controls. Only painful PNP patients showed significantly higher pain ratings and prolonged N1-latencies. PREP-amplitudes did not differ between groups. No significant correlation was found between the average pain intensity during the last 4 weeks and N1-latencies or N1P1-amplitudes.

#### 3.2.3 Comparison between the LFN, MFN, SFN subgroups and healthy controls

MFN patients showed significantly higher DT than healthy controls and SFN patients. PT and stimulation intensities were also higher in MFN compared to healthy controls. MFN and SFN patients reported significantly higher pain ratings at the two-fold PT compared to healthy controls. Only MFN and LFN had significantly prolonged latencies compared to healthy controls and SFN patients. N1P1-amplitudes did not differ between groups. No significant correlation was found between N1-latencies or N1P1-amplitudes and CDT/WDT z-scores. A significant correlation between body height and N1-latencies was only observed when patients with Aß-fiber affection, that is, LFN and MFN, were combined (*r* = 0.465, *p* = 0.013). LFN patients were significantly taller when compared to SFN patients (*p* = 0.049); other group comparisons showed no significant height differences.

### 3.3 CPM

#### 3.3.1 Patients vs. healthy controls

Table 3 presents CPM parameters and significant *p*-values. To account for multiple testing, a Bonferroni correction was applied. Results that remained statistically significant after block wise correction are highlighted in bold in Table 3. Patients reported electrically induced pain of NRS 60 at significantly lower intensities than healthy controls. Pain ratings were lower in patients throughout the procedure, with significant differences at TS_baseline_ and TS_after_. N1P1-amplitudes were significantly reduced in patients compared to healthy controls during CPM.

**Table 3:** CPM parameters.

			Subgroups depending on the presence of pain		Subgroups depending on the affected nerves			
CPM parameters	Healthy controls (*n* = 19)	All patients (*n* = 44)	Painful (*n* = 34)	Painless (*n* = 10)	Painful LFN (*n* = 8)	Painful MFN (*n* = 17)	Painful SFN (*n* = 9)	*p*-values
NRS 60 rated current intensity (mA)	9.77 ± 8.6 (8; 1.2-40)	6.03 ± 5.7 (4.5; 1.3-35)	4.39 ± 2.2 (4.3; 1.3-11.6)	11.47 ± 9.6 (8.5; 3.4-35)	6.24 ± 2.39 (5.7; 4.3-11.6)	3.75 ± 1.51 (3.2; 1.3-6.5)	3.88 ± 2.32 (3.3; 1.4-9)	**AP vs. HC: 0.011,** **MFN vs. HC: <0.001,** MFN vs. LFN: 0.048, **SFN vs. HC: 0.003,** **PF vs. HC: <0.001,** **PF vs. PL: 0.002**
Pain rating baseline (NRS)	65.69 ± 12.12 (67.6; 40.6-82)	56.78 ± 14 (58; 12-83)	54.14 ± 13.4 (53; 12-83)	65.76 ± 12.8 (65; 46-83)	56.3 ± 9.89 (54.5; 43-73)	50.19 ± 15.43 (51; 12-83)	59.67 ± ± 10.15 (59; 45-75)	AP vs. HC: 0.016, **MFN vs. HC: 0.001, PF vs. HC: 0.003,** PF vs. PL: 0.016
Pain rating during CS (NRS)	55 ± 19.84 (56; 24-92)	48.32 ± 14.4 (50; 14-80)	46.62 ± 13.4 (49; 14-68.2)	54.1 ± 17 (55.5; 20-80)	46 ± 15.28 (50; 14-66)	45.6 ± 12.87 (45; 15-68.2)	49.11 ± 13.91 (53; 26-67)	
Pain rating of CS (NRS)	62.63 ± 20.84 (60; 30-100)	59.59 ± 30.2 (67.5; 0-100)	60.35 ± 27.3 (65; 0-100)	57 ± 40.2 (72.5; 0-100)	49.38 ± 30.99 (50; 0-90)	56.29 ± 25.4 (60; 10-90)	77.78 ± 21.23 (85; 50-100)	MFN vs. SFN: 0.041, LFN vs. SFN: 0.027
Pain rating after CS (NRS)	61.19 ± 16.81 (64.6; 26-84)	51.67 ± 16.2 (48.5; 18-90)	49.22 ± 15.4 (47; 18-89)	59.98 ± 16.9 (64; 34-90)	53.68 ± 10.71 (50.5; 40-70)	45.31 ± 17.84 (45; 18-89)	52.67 ± 13.33 (56; 34-76)	AP vs. HC: 0.030, **MFN vs. HC: 0.004, PF vs. HC: 0.01**
N1P1-amplitude baseline (μV)	35.61 ± 14.32 (34.45; 12.39-72.01)	27.64 ± 14.5 (25.72; 9.85-87.68)	28.75 ± 15.8 (25.91; 9.85-87.68)	24.06 ± 8.7 (24.35; 10.76-38)	23.36 ± 13.42 (21.27; 10.84-51.74)	26.56 ± 9.81 (25.91; 9.86-43.46)	36.1 ± 22.36 (32.73; 9.85-87.68)	AP vs. HC: 0.017, **LFN vs. HC: 0.015,** PF vs. HC: 0.039, PL vs. HC: 0.03
N1P1-amplitude during CS (μV)	28.5 ± 10.94 (27.14; 12.83-46.29)	21.64 ± 13 (19.71; 7.53-76.49)	22.06 ± 14.19 (20.7; 7.53-76.49)	20.6 ± 10.4 (18.24; 10.24-42.92)	18.38 ± 8.38 (17.53; 8.09-29.73)	18.8 ± 7.66 (19.23; 7.53-29.83)	29.42 ± 21.8 (23.76; 10.35-76.49)	**AP vs. HC: 0.014,** MFN vs. HC: 0.036, PF vs. HC: 0.029
N1P1-amplitude after CS (μV)	30.18 ± 9.69 (27.58; 17.82-48.9)	24.2 ± 13.3 (21.29; 11.59-88.3)	24.42 ± 14.7 (20.99; 11.59-88.3)	23.56 ± 8.1 (21.75; 13.33-34.87)	19.5 ± 6.62 (19.7; 12.62-31)	22.29 ± 7.89 (21.16; 11.59-41.53)	30.54 ± 22.87 (24.02; 11.84-88.3)	**AP vs. HC: 0.007,** MFN vs. HC: 0.020, LFN vs. HC: 0.014, **PF vs. HC: 0.008**
Parallel CPM-effect_AMPLITUDE_ *n* (%)	17 (89%)	22 (50%)	16 (47%)	6 (60%)	5 (25%)	7 (44%)	5 (56%)	
Parallel CPM-effect_AMPLITUDE_	0.84 ± 0.27 (0.81; 0.55-1.77)	0.82 ± 0.3 (0.79; 0.3-1.54)	0.81 ± 0.3 (0.79; 0.3-1.54)	0.85 ± 0.3 (0.79; 0.46-1.29)	0.84 ± 0.31 (0.94; 0.38-1.23)	0.72 ± 0.34 (0.63; 0.3-1.4)	0.9 ± 0.33 (0.87; 0.54-1.54)	-
Sequential CPM-effect_AMPLITUDE_ *n* (%)	13 (68%)	22 (50%)	18 (53%)	4 (40%)	5 (63%)	7 (44%)	6 (67%)	
Sequential CPM-effect_AMPLITUDE_	0.92 ± 0.29 (0.87; 0.4-1.62)	0.92 ± 0.28 (0.87; 0.5-1.48)	0.88 ± 0.3 (0.83; 0.5-1.48)	1.02 ± 0.3 (1.03; 0.72-1.43)	0.85 ± 0.34 (0.8; 0.5-1.46)	0.9 ± 0.27 (0.87; 0.5-1.32)	0.88 ± 0.29 (0.83; 0.58-1.48)	-
Parallel CPM-effect_PAIN_ *n* (%)	14 (74%)	30 (68%)	23 (68%)	7 (70%)	5 (63%)	11 (69%)	7 (78%)	
Parallel CPM-effect_PAIN_	10.69 ± 15.75 (6.4; 45-18)	8.45 ± 11.65 (8; 44.4 - 14)	7.51 ± 11.68 (7.5; 44.4-14)	11.66 ± 11.5 (13.5; 28-2)	10.3 ± 16.38 (2; 44.4-4)	4.59 ± 10.13 (6; 26-14)	10.56 ± 9.29 (13; 26-4)	-
Sequential CPM-effect_PAIN_ *n* (%)	12 (63%)	31 (70%)	26 (76%)	5 (50%)	6 (75%)	12 (75%)	8 (89%)	
Sequential CPM-effect_PAIN_	4.51 ± 13.58 (2; 37-18)	5.11 ± 11.08 (4; 38-30)	4.91 ± 10.99 (4; 38-30)	5.78 ± 11.9 (3.4; 25-12)	2.63 ± 6.71 (3.5; 12.6-10)	4.88 ± 14.56 (4; 38-30)	7 ± 5 (10; 12-1)	-

Given are the mean value and the standard deviation as well as the median and the minimum to maximum. Only patients with painful PNP were divided into subgroups depending on the affected nerve. To control for multiple comparisons, a Bonferroni correction was applied. The significance level was adjusted to *p* < 0.0167. Results that remained statistically significant after correction are highlighted in bold. LFN, large fiber neuropathy; MFN, mixed fiber neuropathy; SFN, small fiber neuropathy; AP, all patients; HC, healthy controls; PF, painful; PL, painless; DT, detection threshold; PT, pain threshold; NRS, Numeric Rating Scale; CS, conditioned stimulus; CPM, conditioned pain modulation.

**Figure 3:**
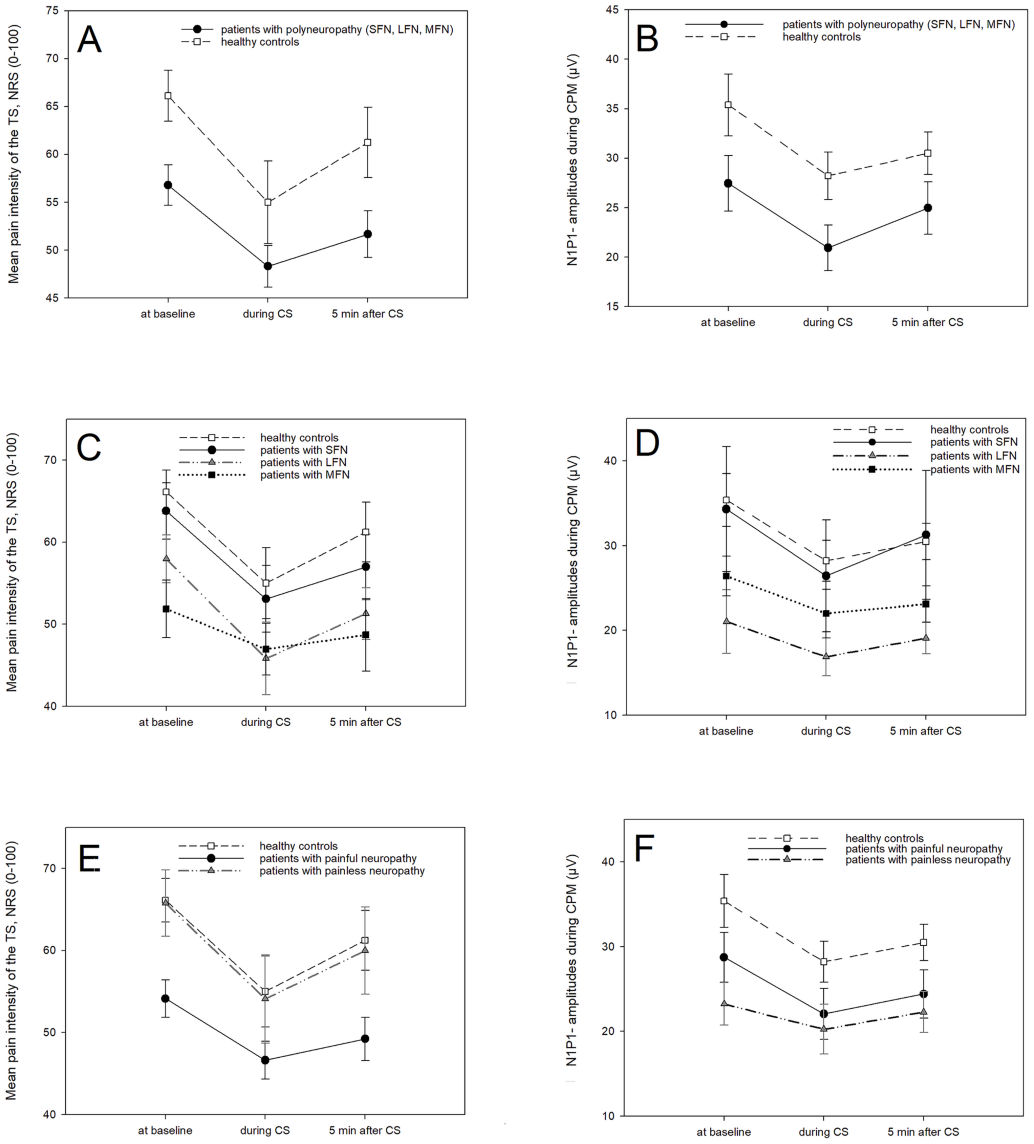
Results of CPM procedure. **(A)** Mean pain intensity of TS in all patients with PNP and healthy controls. **(B)** Mean N1P1-amplitude in all patients with PNP and healthy controls. **(C)** Mean pain intensity of TS in healthy controls, patients with LFN, patients with MFN, and patients with SFN. **(D)** Mean N1P1-amplitude in healthy controls, patients with LFN, patients with MFN, and patients with SFN. **(E)** Mean pain intensity of TS in healthy controls, patients with painful PNP, and patients with painless PNP. **(F)** Mean N1P1-amplitude in healthy controls, patients with painful PNP, and patients with painless PNP. CPM, conditioned pain modulation; PNP, polyneuropathy; LFN, large fiber neuropathy; MFN, mixed fiber neuropathy; SFN, small fiber neuropathy; CS, conditioned stimulus; TS, test stimulus; NRS, Numeric Rating Scale.

Figures 3A,B show CPM results. In both groups, the mean pain intensity of the TS decreased significantly between TS_baseline_ and TS_during_ (healthy controls: *p* = 0.004, patients: *p* < 0.001). In patients, TS_after_ was also significantly lower than TS_baseline_ (*p* = 0.019). In healthy controls, N1P1-amplitudes decreased significantly during CS application compared to baseline (*p* < 0.001) and after CS (*p* = 0.035). Similarly, in patients, the N1P1-amplitudes were also significantly reduced during CS application compared to baseline (*p* < 0.001) and after CS (*p* = 0.002). We found no significant differences in CPM-effects. CPM-effects did not correlate with disease duration, PREP N1-latencies/N1P1-amplitude, or painfulness of PREP.

#### 3.3.2 Comparison between the painful, painless subgroup and healthy controls

Patients with pain reported electrically induced pain of NRS 60 at significantly lower intensities than healthy controls and painless patients. Pain ratings for TS_baseline_ and TS_after_ were significantly lower in painful PNP compared to healthy controls; TS_baseline_ was also lower compared to painless PNP. Both patient groups showed significantly reduced N1P1-amplitudes at baseline compared to healthy controls. N1P1-amplitudes during and after CS application were only significantly reduced in painful PNP compared to healthy controls. No significant CPM-effects and no correlation between CPM-effects and the average pain intensity during the last 4 weeks were found.

Figures 3E,F show CPM results. In painful PNP, the mean pain intensity of the TS decreased significantly between baseline and during (*p* < 0.001) and after (*p* = 0.025). In painless PNP, the mean pain intensity of the TS did not change significantly. In patients with painful PNP, the N1P1-amplitudes decreased significantly between baseline and during (*p* = 0.002).

#### 3.3.3 Comparison between the painful LFN, painful MFN, painful SFN subgroups and healthy controls

For a detailed analysis of the CPM, only patients with pain were divided into subgroups (painful LFN, painful MFN, painful SFN).

Patients with painful MFN and SFN reported electrically induced pain of NRS 60 at significantly lower intensities than healthy controls; painful MFN patients also differed significantly from painful LFN. Painful MFN patients showed significantly lower pain ratings for TS_baseline_ and TS_after_ compared to healthy controls. Pain ratings for the cold-water bath were significantly lower in painful LFN and MFN than in SFN but did not differ from healthy controls. N1P1-amplitudes of the TS at baseline were significantly reduced in painful LFN patients compared to healthy controls. During CS, N1P1-amplitudes were significantly lower in painful MFN; after CS, they were lower in both painful LFN and MFN compared to healthy controls. There were no significant differences in CPM-effects.

Figures 3C,D shows CPM results. The mean pain intensity of the TS did not change significantly in painful LFN and MFN patients. Only painful SFN patients showed a significant reduction of the mean pain intensity of the TS between baseline and during CS application (*p* = 0.01). N1P1-amplitudes remained stable in painful MFN and SFN; in LFN, they decreased significantly during CS application compared to baseline (*p* = 0.027).

## 4 Discussion

### 4.1 Nociception measured by PREP

This study was the first to investigate CPM-effects in PNP patients based on nerve fiber involvement and pain presence, using a protocol with electrical stimulation via concentric electrode as TS and calculating the CPM-effects based on changes in objective and subjective outcomes. There were no significant differences in CPM-effects between the groups. However, painful PNP – especially with small fiber involvement – affected PREP and CPM parameters, likely due to sensitization and deafferentation.

As a sign of deafferentation, patients showed significantly higher DT and PT compared to healthy controls. Although stimulation intensities were comparable between patients with SFN and healthy controls, individuals with SFN reported significantly greater pain ratings. However, this finding does not clearly demonstrate deafferentation, suggesting that any underlying sensory loss may not yet be pronounced enough to produce measurable changes in pain thresholds. Nevertheless, the condition appears to be clinically relevant, as increasing stimulation intensities eventually provoked greater pain responses.

Consistent with previous research (12, 13, 15, 28, 39, 40), we observed prolonged N1-latencies in patients compared to healthy controls, but only in patients with MFN and LFN. Patients with SFN showed latencies comparable to those of healthy controls. In a similar study by Siedler et al. (28), no prolonged latencies were found in patients with LFN, despite reduced IENFD in skin biopsies in 65% of those patients. In contrast, our patients with LFN demonstrated the longest latencies. Although it was not possible to classify the subgroups based on recommended criteria, such as the ACTTION criteria (41), the cohort was distributed based on established clinical tests, such as NCS and QST as well as skin biopsy if available. However, a subtle involvement of small fibers cannot be excluded. In our study, a subclinical impairment of small fibers that is not detectable by QST remains possible even though a recently published paper once again demonstrated the reliability of QST in the detection of small fiber impairment (42). Furthermore, it is important to critically evaluate whether PREP exclusively reflects Aδ- and C-fiber pathways. The literature has repeatedly questioned whether concentric surface electrodes selectively stimulate small fibers or whether large myelinated Aß-fibers may also be activated (43, 44). An alternative explanation for the differences in latencies may be the body height. While patients with SFN and healthy controls had similar mean heights, patients with LFN were slightly taller. We additionally observed a positive correlation between body height and N1-latency. Previous studies have already identified the distance between the stimulation site and the recording electrode as a relevant influencing factor (33, 45).

Unlike previous studies (11–14, 46), we found no significantly reduced PREP amplitudes. This may underlie different pathophysiological mechanisms. Patients required significantly higher stimulation intensities than healthy controls. This finding suggests that peripheral deafferentation may be partially compensated by stronger nociceptive stimulation, resulting in comparable amplitude levels between groups. For example, Mueller et al. (12) limited stimulation to 2.4 mA in their study – this cut-off is lower than the mean stimulation intensity in our study – and reported reduced amplitudes, possibly due to lower stimulation. This raises the possibility that lower stimulation intensities contributed to the observed amplitude reduction. In the other studies mentioned above (13, 14, 46) it was only stated that stimulation was applied at twice the individually determined PT, similar to our approach. However, it remains unclear whether reduced amplitudes in those studies may also have been influenced by comparatively lower stimulation intensities.

In our cohort, patients with both painful and painless PNP required higher stimulation intensities compared to healthy controls. However, only patients with painful PNP rated the stimuli as significantly more painful. Forstenpointner et al. (7) examined QST in patients with painful and painless PNP as well as in healthy controls; interestingly reduced pain thresholds were observed in both patient groups. Nevertheless, self-reported pain sensitivity was significantly higher in patients with painful PNP compared to patients with painless PNP. This suggests that mechanisms beyond structural nerve damage may contribute to pain perception, potentially reflecting the sensitization process. Prolonged latencies were observed exclusively in the painful subgroup, indicating a possible association between pain and latency. To date, there are no studies that have compared PREPs between patients with painful and painless PNP. A negative correlation between burning pain and PREP amplitude was reported in a study that investigated PREPs in patients with painful MFN (27). Interestingly, we did not detect significant amplitude differences between painful and painless patients in our cohort.

### 4.2 Endogenous pain modulation

Similar to PREP findings, the CPM assessments also revealed signs of sensitization. Patients with painful PNP – particularly those with painful MFN or SFN – reported electrically induced pain of NRS 60 at significantly lower intensities than healthy controls and painful LFN patients. After TS_baseline_, patients rated the TS as less painful than healthy controls, particularly in painful PNP. This phenomenon may result from preexisting sensitization or anxiety in patients, which may have led to the TS initially being rated as more painful.

Yarnitsky et al. (14) investigated the effects of duloxetine in patients with painful diabetic PNP and demonstrated that a reduced CPM-effect was associated with superior therapeutic response compared to patients showing a normal CPM-effect. This finding was interpreted as evidence of dysfunctional central pain processing, which can be modulated by serotonergic-noradrenergic agents such as duloxetine (47). In our cohort, comparable signs of central sensitization were observed, for example, reflected by increased pain perception at lower stimulation intensities.

N1P1-amplitudes generated by the TS were reduced in patients compared to healthy controls. While higher TS intensities increased amplitudes in healthy controls, this was not seen in patients – except in painful SFN patients, possibly due to central sensitization or less detectable deafferentation. Due to concurrent effects of the deafferentation and central sensitization, an interpretation of the amplitudes and especially the CPM-effect_AMPLITUDE_ is challenging.

As previous studies showed reduced CPM-effects in chronic pain (22, 48, 49), we expected similar findings in patients with nociceptive fiber involvement, especially with neuropathic pain. However, no significant differences in CPM-effects were found between patients and healthy controls, suggesting preserved endogenous pain modulation in PNP patients. Contrary to our hypothesis, neither Aδ- and C-fiber damage nor chronic neuropathic pain was linked to reduced CPM-effect. Interestingly, the parallel CPM-effect_PAIN_ was more pronounced in SFN and patients with painful PNP, while the sequential CPM-effect_PAIN_ was only significant in patients, especially those with painful PNP.

To date, only a few studies have examined CPM in patients with PNP. Granovsky et al. (24) reported that CPM efficiency was lower in patients with painless diabetic PNP compared to those with painful diabetic PNP. The authors attributed these findings to potential alterations in central inhibitory pain pathways as well as to stimulus placement. Nahman-Averbuch et al. (25) assessed pain modulation in patients with chemotherapy-induced PNP. Participants were stratified into painful and painless subgroups according to their NRS scores. In their protocol, heat stimuli were used as TS and a cold-water bath served as CS. No significant differences in CPM responses were observed between the two patient groups. However, the study did not include a group of healthy controls for comparison. In a further study that examined the effect of Tapentadol on descending pain inhibition in patients with painful PNP, none of the patients had a proper CPM response before receiving the treatment with Tapentadol. Not only was the relief of pain greater in patients after treatment, but also a significant CPM response was shown (26).

### 4.3 Limitations

The main limitation of the study is the sample size and especially the uneven distribution among the subgroups. Strict exclusion criteria made it difficult to recruit age-matched healthy controls, leading to some demographic differences, but also reducing cofounding factors. Due to different etiologies, the patient collective was heterogeneous. Not all patients received a skin punch biopsy, which may have potentially influenced subgroup allocation. During the CPM procedure, only patients with pain were divided into subgroups based on fiber types. This was due to the small sample size and the small number of painless patients. Unfortunately, a statistical analysis of the interaction between these two factors (painful/painless and small /mixed/large fiber impairment) was not possible. Studies with larger sample sizes are required to explore this in more detail. PREP can be influenced by factors like attention, emotions, smoking, or caffeine (50, 51), and varying stimulation paradigms limit comparability across studies (16). Another aspect that should not be overlooked is the use of medication known to influence PREP responses. As shown in Table 1, most patients did not take any medication. However, a few patients did, and therefore, a potential medication effect cannot be ruled out with certainty. As already mentioned above, higher stimulation intensities may also activate deeper axons (e.g., Aβ-fibers) and cause spatial summation, potentially influencing results. This is particularly relevant in CPM due to the high stimulation intensities involved. However, if the stimulation intensity is too low, it may not elicit a sufficiently strong pain stimulus to induce CPM.

CPM can be conducted in various ways; we used 10 °C cold water as CS. Due to individual differences in temperature sensitivity, for some individuals the inhibitory effect may have been insufficient to reveal group differences. We performed PCES on the upper limb because this area is less frequently affected of PNP symptoms. However, the use of both TS and CS on the upper arm could have influenced the CPM-effect by introducing additional segmental phenomena. We did not include a control group to assess habituation, so its influence remains unclear. However, a prior study suggests that habituation alone cannot explain CPM differences (34). Still, as this previous study focused on healthy subjects, future research should assess habituation effects also in patients to further elucidate the genuine CPM-effect. Similar to other studies (17, 19, 20), we regarded a CPM-effect_PAIN_ < 0 as a hint for efficient pain modulation. However, when interpreting the results, it must also be taken in account that natural variability cannot be ruled out if the TS is applied repeatedly.

Previous studies showed that CPM is less effective in women and elderly people (52, 53). While we found no significant differences in the CPM-effect between the groups. However, a detectable CPM-effect (CPM-effect_AMPLITUDE_ < 1, CPM-effect_PAIN_ < 0) was more frequent in healthy controls than in patients (see Table 3). The presence or absence of pain and/or sensory abnormalities might contribute to this difference. Further, the significant age difference between patients and healthy controls may also have an impact.

Although PNP-related changes like prolonged latencies, deafferentation, and signs of central sensitizations were observed, peripheral nerve damage or chronic neuropathic pain do not appear to affect the CPM-effect itself.

## Data Availability

The raw data supporting the conclusions of this article will be made available by the authors, without undue reservation.

## Author’s note

This work is part of the doctoral thesis of Laura Josephine Bubenzer.
